# Translating Comprehensive Conservative Care for Chronic Knee Pain Into a Digital Care Pathway: 12-Week and 6-Month Outcomes for the Hinge Health Program

**DOI:** 10.2196/rehab.7258

**Published:** 2017-04-05

**Authors:** Peter Smittenaar, Jennifer C Erhart-Hledik, Rose Kinsella, Simon Hunter, Gabriel Mecklenburg, Daniel Perez

**Affiliations:** ^1^ Hinge Health Inc San Francisco, CA United States; ^2^ Department of Orthopaedic Surgery Stanford University Stanford, CA United States

**Keywords:** chronic pain, osteoarthritis, knee, digital health, conservative management

## Abstract

**Background:**

Chronic knee pain (CKP) affects a large number of adults, many of whom do not receive best-practice care and are at high risk for unnecessary surgery.

**Objective:**

The aim of this study was to investigate the effect of the Hinge Health 12-week digital care program (DCP) for CKP on knee pain and function, with secondary outcomes of surgery interest and satisfaction, at 12 weeks and 6 months after starting the program.

**Methods:**

Individuals with CKP were recruited onto the 12-week program, comprising sensor-guided physical exercises, weekly education, activity tracking, and psychosocial support such as personal coaching and cognitive behavioral therapy (CBT). We used a single-arm design with assessment of outcomes at baseline, 12 weeks, and 6 months after starting the program. We used a linear mixed effects model with Tukey contrasts to compare timepoints and report intention-to-treat statistics with last observation carried forward.

**Results:**

The cohort consisted of 41 individuals (32 female, mean age 52 years, SD 9 years). Between baseline and week 12, participants reported clinically significant improvements in the Knee Injury and Osteoarthritis Outcome Score (KOOS) pain and Knee Injury and Osteoarthritis Outcome Score-Physical Function Short Form (KOOS-PS) function scales of 16 points (95% CI 12-21, *P*<.001) and 10 points (95% CI 6-14, *P*<.001), respectively. Significant reductions of 57% (mean difference 30, 95% CI 21-38, *P*<.001) and 51% (mean difference 25, 95% CI 16-33, *P*<.001) in visual analog scale (VAS) knee pain and stiffness, respectively, were observed at 12 weeks, as well as a 67% reduction in surgery interest (mean reduction 2.3 out of 10, 95% CI 1.5-3.1, *P*<.001). Average satisfaction at week 12 was 9.2 out of 10. Critically, all improvements were maintained at 6 months at similar or greater magnitude.

**Conclusions:**

Participants on the Hinge Health DCP for CKP showed substantial clinical improvements that were maintained 6 months after enrolling in the program. This shows that DCPs carry strong potential to deliver evidence-based, cost-effective care to those suffering from CKP.

## Introduction

### Background

Chronic knee pain (CKP) is one of the most common health conditions [1] and is a characteristic presenting symptom of knee osteoarthritis (OA) [2]. People living with CKP experience a reduced quality of life [3] and are at risk of developing concomitant musculoskeletal and mental health conditions [4,5]. CKP is most effectively treated by comprehensive chronic pain programs, comprising not only physical exercise but also education, psychosocial support, and weight loss [6-9]. Such programs have shown clinically relevant reductions in pain that last up to 5 years [10,11] and medical cost savings due to a reduced need for injections, drugs, and surgery [8], with one intervention for CKP due to knee OA showing a 75% (8/41 had knee replacement in control vs 2/42 in treatment) reduction in rate of total knee replacements [12]. Comprehensive care for CKP due to knee OA is also more effective at reducing pain in the long-term compared with physical therapy only [13-16]. However, chronic pain programs are rare for CKP, and over 80% of individuals with CKP due to knee OA receive suboptimal conservative care [17]. Furthermore, CKP patients show poor adherence to existing treatments [18].

The lack of widespread best-practice conservative care for those suffering from CKP drives patients toward total knee arthroplasty (TKA), an expensive intervention which almost doubled in rate between 2000 and 2010 in the United States [19]. Further exacerbated by an aging population, TKAs now represent one of the main cost drivers for self-insured employers and the largest in-patient cost for Medicare, alongside hip replacements. Despite the popularity of the procedure, many patients undergoing TKA may have avoided or at least delayed surgery through comprehensive conservative care [12], with 34% of TKAs performed in the United States regarded as inappropriate [20]. For those that do undergo TKA, the benefits are partly offset by serious adverse events [21,22]. Even more wasteful are arthroscopic debridement surgeries, which have no discernible effect on the patient beyond placebo yet remain one of the most common interventions with 500,000 procedures every year in the United States alone [23]. As such, there is huge scope for effective nonsurgical treatment solutions to improve patient outcomes and drive down the surging costs associated with CKP.

A digital care program (DCP), whereby each facet of evidence-based care is digitized, aims to deliver care more efficiently, effectively, and in a way that would improve outcomes while decreasing costs. In particular, a DCP for CKP administered remotely would allow patients access to the program at any time and place, provide a single touchpoint for every aspect of care, enable rich data collection on patient behavior and progress, and drastically reduce the marginal cost of additional patients receiving treatment. Furthermore, as poor adherence can limit long-term effectiveness of a program for CKP [18], a DCP incorporating remote sensing would enable very precise monitoring of adherence levels to exercise therapy, affording personalized and timely interventions during the course of treatment. Digital health is moving into many different domains of health care, ranging from cognitive behavioral therapy (CBT) for pain and depression to remote monitoring of heart patients [24-26]. In diabetes prevention, a digital health program has shown positive outcomes that persisted up to 2 years after completion of the program [27], and a digital sleep therapy program was found to be effective in a randomized controlled trial [28]. However, the musculoskeletal field has seen relatively little digital innovation and was judged to be “in its infancy” in this regard [29].

The American College of Rheumatology recommends those suffering from CKP to participate in cardiovascular and strengthening exercise, self-management training, psychosocial intervention, and weight loss for overweight patients [7]. In line with these recommendations, we have developed a 12-week DCP for CKP. The program builds on previous work in digital musculoskeletal care, which studied individual components of digital care in isolation, such as diagnosis [30], CBT [25], exercise with telephone-based coaching [31], exercise with pain coping training [32], and behavioral change approaches [33].

### Aims of This Study

The aims of this study were to (1) determine the change in pain and function between baseline and follow-up (week 12 and 6 months) in participants in the 12-week Hinge Health DCP and (2) assess changes in surgery interest and patient satisfaction between baseline and follow-up.

## Methods

### Research Design

We used a single-arm design with patient-reported outcome measures (PROMs) collected before starting the program (“baseline”), at the end of the 12-week program, and at 6 months after starting the program.

### Participants

The 12-week Hinge Health DCP was deployed at two sites in the United States, both of which compensated Hinge Health for the deployment. All potential participants were employees of a self-insured employer, covered by their medical plan. Potential participants were recruited by email, letters mailed to their home address, and fliers posted in the workplace, and were screened for inclusion by Web-based questionnaire. For inclusion, subjects had to provide written informed consent, have lived with knee pain for at least 3 months in the past 12 months, and had to meet at least 2 of the following additional inclusion criteria derived from the American College of Rheumatology criteria for OA of the knee [2]: morning stiffness lasting less than 30 min, crepitus on movement, bony tenderness, bony enlargements, lack of warmth of the knee to the touch, and age of 50 years or older. Exclusion criteria were knee surgery or trauma in the past 3 months. We obtained ethical approval to conduct a research study as part of these deployments from the Western Institutional Review Board (WIRB 20160949).

An a priori sample size calculation was performed for comparing the primary outcomes of pain and function. Using an alpha level of .05, a power of 0.8, and a medium effect size of 0.5, 33 subjects were needed. Recruitment of 41 participants accounted for a potential dropout rate of 20% over the course of the study. As there were a limited number of places available on the program, we invited eligible applicants on a first come, first serve basis. Users were not compensated for their time, but could participate in the program free of charge.

### Intervention

**Figure 1:**
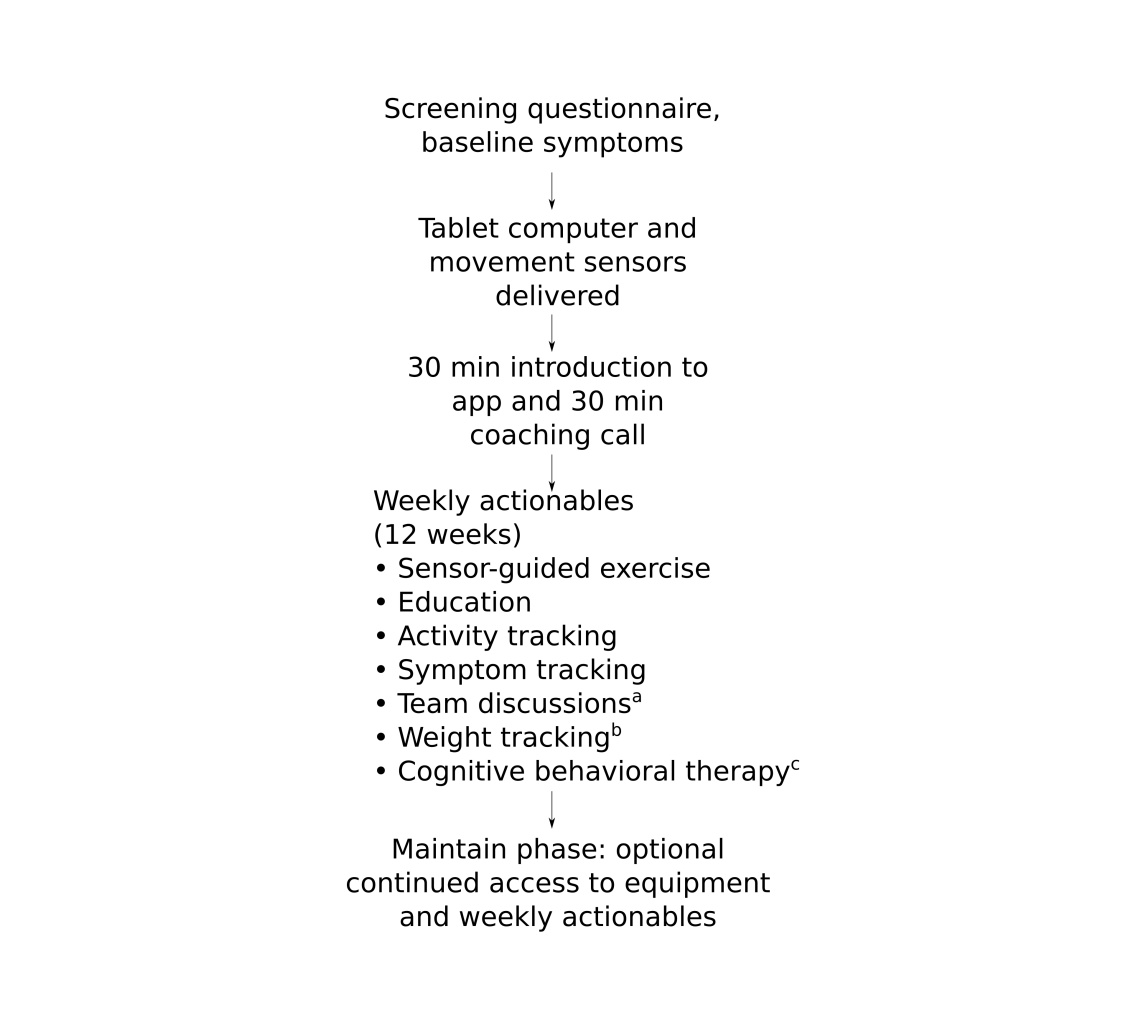
User flow in the Hinge Health digital care program. (a) Every odd-numbered week. (b) Only for those with a starting body mass index (BMI) of 25 kg/m2 or greater. (c) Only on a subset of weeks and only for those users who qualified for the respective cognitive behavioral therapy module (see "Methods" section).

**Figure 2:**
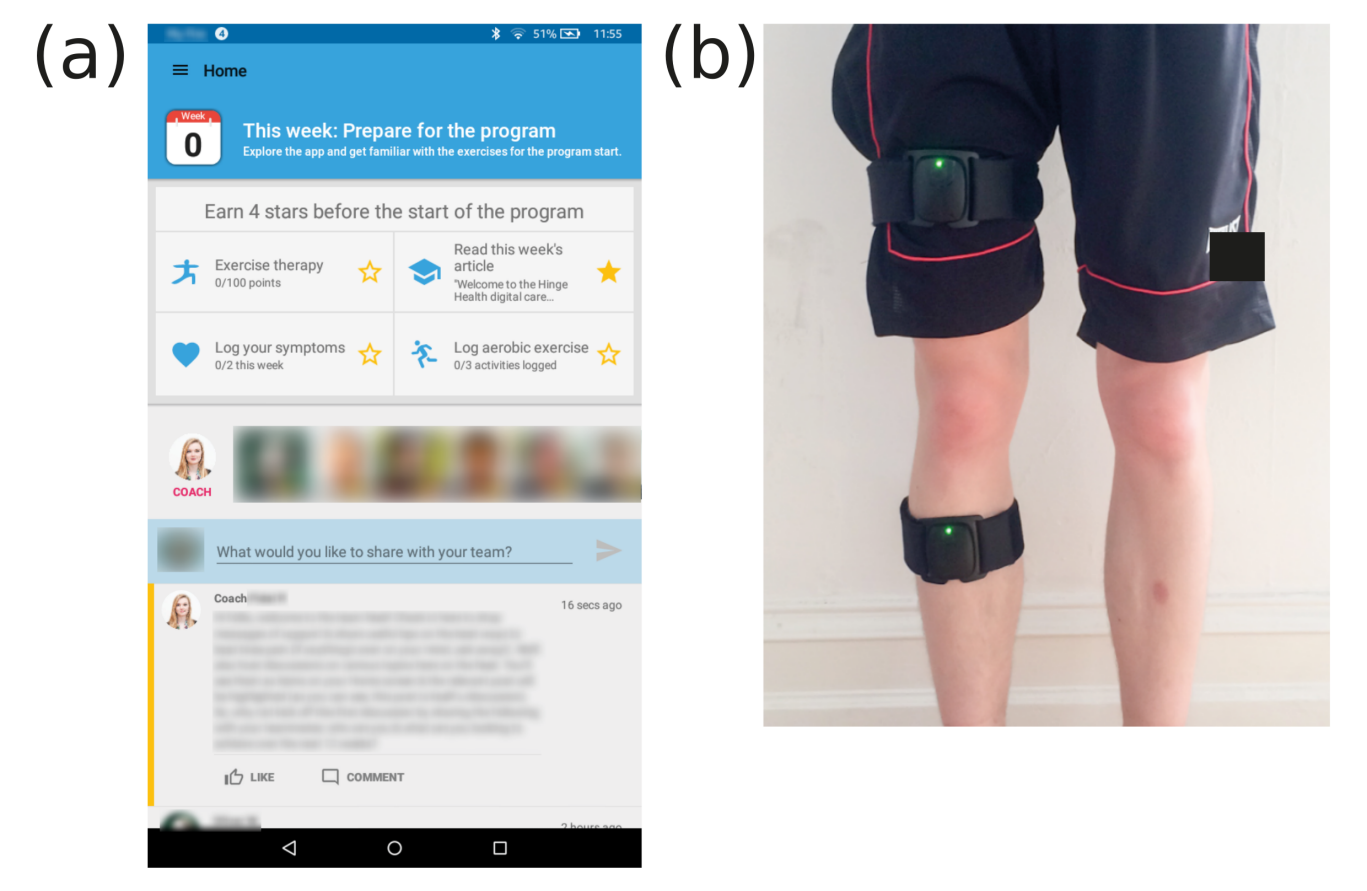
Tablet computer and sensors as part of the Hinge Health kit. (a) A screenshot of the home screen. Weekly actionables are indicated by stars, followed by an overview of fellow team members and the team discussion feed. Further functionality—including a progress screen, education articles, and private communication channel with the coach—are available through the menu. (b) Placement of sensors for exercise therapy.

The Hinge Health DCP is a 12-week program (Figure 1) which aims to equip participants with the knowledge and tools to self-manage their condition without prescription drugs and surgery as long as possible. The program comprises sensor-guided physical exercise, education, CBT, psychosocial support through teams and personal health coaches, weight loss, and activity tracking. In the week before the official start of the program, each invited participant was assigned to a team of 15-20 participants and taken through a 30-min in-person onboarding session led by a trained Hinge Health representative. During this session, the participant was provided with a tablet computer preloaded with the Hinge Health app as well as wearable bands with motion sensors to be used during guided exercises (Figure 2), and shown how to use the main features of the app and perform sensor-guided exercise therapy. This was followed within a few days by a 30-min call with a personal coach, who was an employee of Hinge Health trained for interaction with participants. The purpose of the call was for the coach to establish themselves as the primary touchpoint for the participant throughout the program, orient the participant to the program, help set goals, and identify and alleviate practical barriers to adherence. Every week on the program participants had to complete a number of goals. These components of the program are discussed below. Participants were allowed to keep their tablet computer and movement sensors after completion of the 12-week program, and they could continue to interact with the program as desired to access education, communicate with teammates, log symptoms, and track activities; however, no activities were required of participants during this maintenance phase.

### Exercise Therapy

Participants had a weekly exercise repetitions goal for sensor-guided exercises, which increased over the course of the program. Approximately 15 min of stretching and strengthening exercise for 3-4 days per week was sufficient to reach their weekly goal. Specifically, we provided the following sensor-guided exercises: standing quad stretch (pulling heel toward buttocks), seated quad stretch (pull leg toward chest), half squats, forward lunges, leg raise (raising lower leg behind the body until parallel with floor while holding chair), seated leg raise (raising lower leg to horizontal while seated), and hamstring stretch (foot on raised object, reach to touch toes with straight leg). The app tracked the execution of the exercises and provided real-time feedback to the user to ensure that the exercises were performed correctly. Before starting a new exercise, a narrated video showed correct execution, and this video remained available to the participant throughout the program. Crucially, the sensors afforded an objective avenue to monitor adherence.

### Education

Education articles were presented once per week, for a total of 12 education articles, each requiring approximately 10-20 min of reading. Each article consisted of approximately 6 pages, and we tracked consumption of each page. A piece of education was marked complete if the participant reached the final page of the article.

### Symptom Logging

Participants were asked to log their pain and stiffness symptoms on a visual analog scale (VAS) at least twice a week, alongside any treatments they had been using for their knee. Participants were prompted to fill out questionnaires at predetermined timepoints in order to track PROMs. The specific timepoints for each PROM are outlined below.

#### Activity Tracking

A self-report activity tracker helped log any physical activity they performed during the week, encouraging at least three 30-min sessions per week of low-impact exercise.

#### Cognitive Behavioral Therapy

CBT modules were provided. One was provided to all users (*pacing activity levels*), whereas others were provided based on data provided by users: the *weight loss* CBT for participants with a body mass index >25 kg/m^2^; the *coping with pain* CBT for users with a score greater than 30 on the pain catastrophizing scale; the *low mood* and *anxiety* CBT for participants with a score of 10 or higher on the Hospital Anxiety and Depression Scale (HADS), respectively.

#### Team and Coach Interaction

The coach facilitated in-app team discussions, while encouraging team members to discuss anything of interest with their teammates on the team feed (accessible via the app). Participants communicated with the coach through the tablet app, phone, SMS, or email. The participants could initiate a conversation at any time and the coach would respond within the same day. Moreover, the coach sent weekly messages to introduce the week’s education, provide feedback on completed CBT modules, send an overview of the participant’s performance in the previous week, and encourage the user to attend to their weekly goals on Wednesdays and Fridays, if the participant was behind on their goals.

### Primary Outcomes: Pain and Function

We used the Knee Injury and Osteoarthritis Outcome Score (KOOS) 9-question pain subscale [34,35], as well as the 7-question Knee Injury and Osteoarthritis Outcome Score-Physical Function Short Form (KOOS-PS) to assess function [36]. KOOS questionnaires were asked at baseline (screening) as well as at week 4, 8, and 12 of the program, and scored from 0 (no symptoms) to 100 (extreme symptoms). Both questionnaires were also administered at the 6-month timepoint.

### Secondary Outcomes

Participants reported on their knee pain and function by completing VAS questions at baseline (screening) and twice per week during the program, asking “Over the past 24 h how bad was your knee pain?” and “Over the past 24 h how bad was your knee stiffness?” respectively. The left pole was set to 0 and contained the text “none,” and the right pole was set to 100 and contained the text “worst imaginable.” Unlike other PROMs, VAS reports were optional in the app. To assess overall satisfaction with the program, we asked “On a scale of 0-10, how likely is it that you would recommend the Hinge Health program to a friend or colleague?” at week 6 and week 12. We tracked participants’ self-reported likelihood of undergoing knee surgery at baseline (screening), week 6, and week 12 of the program by asking “On a scale from 0 to 10, how interested are you in knee surgery?” All secondary outcomes were also assessed at 6-month timepoint.

### Statistical Analysis

We report *intention-to-treat* statistics with last observation carried forward. We used a linear mixed effects model implemented through LME4 [37] and implemented Tukey contrasts to compare timepoints through the “multcomp” package [38] in the statistical computing software R (version 3.3.2, The R Project for Statistical Computing). We modeled a single within-subject factor “time” (levels: baseline, 12 weeks, 6 months), and a separate baseline for each participant. We modeled time as a categorical factor and therefore do not assume a linear relationship between time and outcome measures. We report the contrast estimate, 95% CI on the estimate, and *P* value. *P* values <.05 were considered significant. We also examined the *per protocol* results. Due to the low dropout rate, these results were not meaningfully different from the *intention-to-treat* results and are therefore not reported here.

## Results

### Participants

Demographics of participants are presented in Table 1. On average, participants were aged above 50 years, had a BMI over 25 kg/m^2^, and predominantly female. At baseline, 66% (27/41) of users were not doing any physical therapy-style exercise and 54% (22/41) were active 90 min or less per week including walking, suggesting a predominantly sedentary lifestyle. There were no significant differences in any of the demographics or baseline data between those who completed the PROMs at 6 months and those who did not (*P*>.05 for all).

**Table 1:** Demographics and relevant baseline data.

Metric	All Participants	Completed 12 week PROMs^a^	Completed 6 month PROMs	Did not complete 12 week	Did not complete 6 month
n (% of all participants)	41 (100)	37 (90)	33 (80)	4 (10)	8 (20)
Age in years, mean (SD^b^)	52 (9)	52 (9)	54 (8)	54 (4)	47 (9)
BMI^c^ (kg/m^2^), mean (SD)	29 (7)	28 (7)	29 (7)	32 (6)	27 (7)
Height (cm), mean (SD)	169 (10)	169 (10)	168 (8)	171 (4)	176 (13)
Weight (kg), mean (SD)	82 (17)	80 (17)	81 (17)	92 (15)	83 (19)
Female, n (%)	32 (78)	29 (78)	28 (85)	3 (75)	4 (50)
PT-like exercise^d^ at baseline, n (%)	14 (34)	13 (35)	11 (33)	1 (25)	3 (38)
Active 90+ min per week at baseline, n (%)	19 (46)	19 (51)	18 (55)	0 (0)	1 (12)
Pain catastrophizing scale^e^, mean (SD)	14 (9)	13 (10)	13 (10)	19 (5)	16 (8)
Had knee surgery in past, n (%)	17 (41)	15 (41)	15 (45)	2 (50)	2 (25)
Arthritis diagnosed by doctor, n (%)	18 (44)	17 (46)	17 (52)	1 (25)	1 (12)

^a^PROMs: patient-reported outcome measures.

^b^SD: standard deviation.

^c^BMI: body mass index.

^d^PT-like exercise: answer to screening question “Do you currently do any physical therapy-style exercises?”

^e^Pain catastrophizing scale: from 0 (no catastrophizing) to 52 (extreme).

### Intervention Engagement

Engagement across each of the relevant goals provided to participants in the program are shown in Table 2. Participants performed sensor-guided physical exercises on 42.9 days on average, or 3.6 days per week—in line with the goal of 3-4 days exercise per week. On such an average active day, participants performed 39 repetitions across various exercises. Participants also completed the majority of their education articles, consuming education on 89% (10.7/12) of weeks. The average participant completed 1.9 (SD 0.8) of the 3.3 (SD 0.8) CBT sessions offered.

### Primary Outcomes: Pain and Function

**Figure 3:**
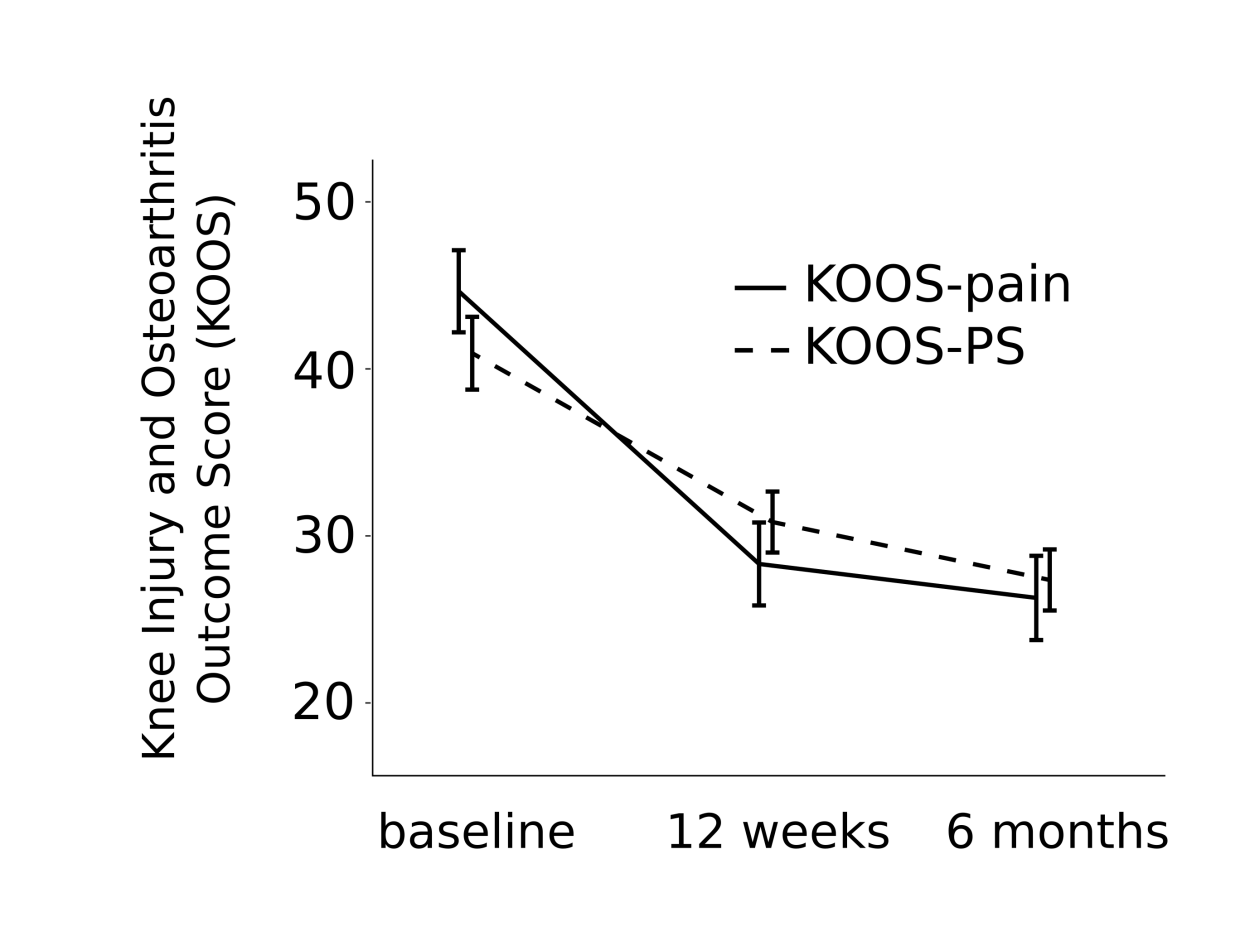
Knee Injury and Osteoarthritis Outcome Score (KOOS) pain subscale and Knee Injury and Osteoarthritis Outcome Score-Physical Function Short Form (KOOS-PS)—which measures knee function—over the course of the 6-month assessment period. Error bars indicate standard error of the mean (SEM).

Participants reported highly significant improvements on the KOOS pain subscale (Figure 3; improvement at week 12 from baseline: 16 points, 95% CI 12-21, *P*<.001) that were maintained at 6 months (improvement from baseline: 18 points, 95% CI 14-23, *P*<.001). Knee function also significantly improved at 12 weeks (KOOS-PS, Figure 3; improvement at week 12 from baseline: 10 points, 95% CI 6-14, *P*<.001) and was maintained at 6 months (improvement from baseline: 14 points, 95% CI 9-18, *P*<.001).

**Table 2:** Engagement with the Hinge Health digital care program (DCP) for chronic knee pain (CKP).

Metric	All Participants	Completed 12 week PROMs^a^	Completed 6 month PROMs	Did not complete 12 week	Did not complete 6 month
Days with sensor-guided exercise, mean (SD^b^)	42.9 (16.1)	44.8 (15.2)	46.7 (14.5)	26 (16.1)	27.4 (13.3)
In-app physical exercise repetitions, mean (SD)	1685.5 (1150)	1772.6 (1163.1)	1881.2 (1175)	880.2 (665.1)	878.1 (565.8)
Offline activities logged in hours, mean (SD)	24.9 (11.5)	26 (11.4)	27.2 (11.3)	14.8 (5.8)	15.4 (5.8)
Education articles read, mean (SD)	10.7 (2.1)	10.9 (1.6)	11.1 (1.5)	8.5 (4.4)	8.9 (3.1)
CBT^c^ session completed, mean (SD)	1.9 (0.8)	1.9 (0.8)	2 (0.7)	1.5 (1)	1.4 (1.1)
Team posts and comments, mean (SD)	12.3 (7.7)	12.9 (7.7)	13.9 (7.4)	6.2 (3.2)	5.4 (3.9)

^a^PROM: patient-reported outcome measure.

^b^SD: standard deviation.

^c^CBT: cognitive behavioral therapy.

### Secondary Outcomes

#### Visual Analog Scales

**Figure 4:**
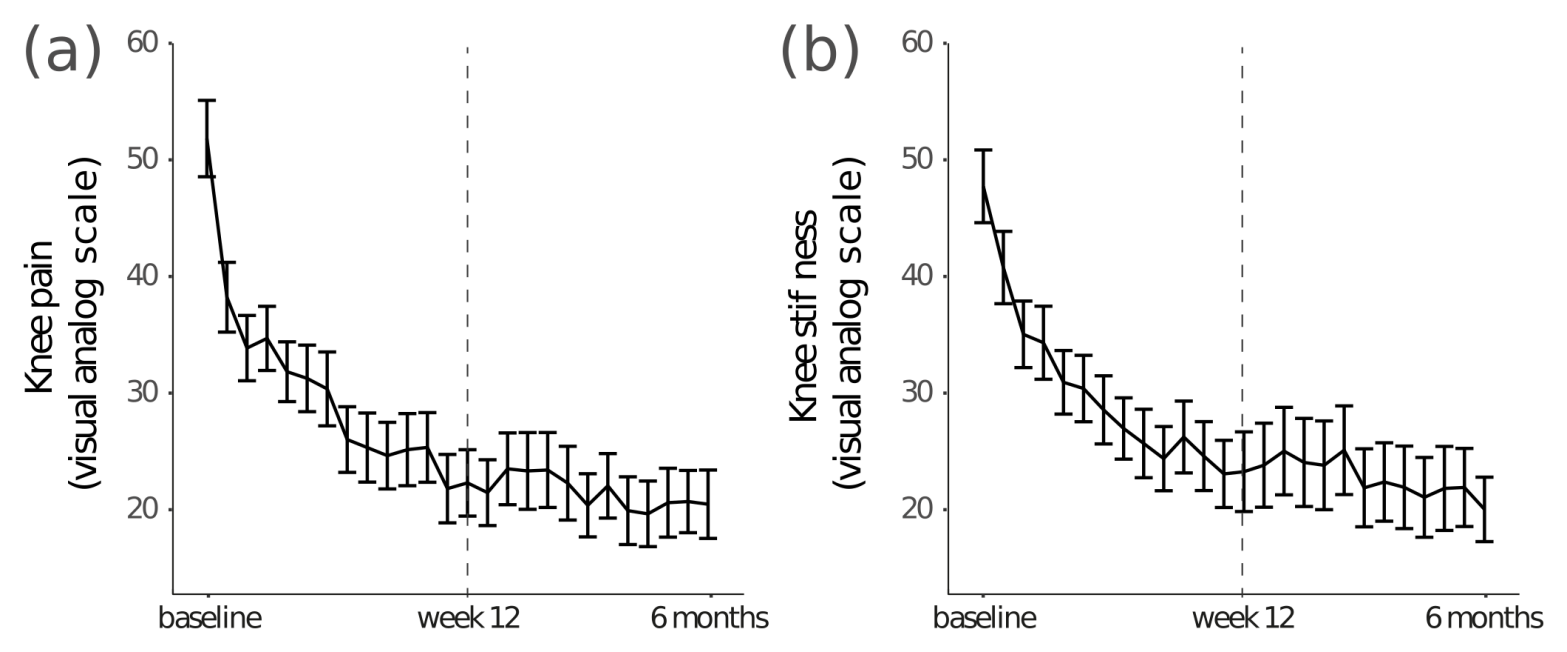
Visual analog scale assessment of (a) knee pain and (b) knee stiffness over the course of the 6-month assessment period. The dotted line indicates the last week of the 12-week program. Error bars indicate standard error of the mean (SEM).

Between baseline and week 12, participants reported a 57% reduction in knee pain (Figure 4; from 52 to 22 points; mean difference 30, 95% CI 21-38, *P*<.001) and 51% reduction in knee stiffness (Figure 4; from 48 to 23; mean difference 25, 95% CI 16-33, *P*<.001). These improvements were maintained at 6 months for both knee pain (mean improvement 31, 95% CI 23-40, *P*<.001) and stiffness (mean improvement 28, 95% CI 20-36, *P*<.001).

#### Surgery Intent

Surgery interest significantly decreased over the course of the program from 3.5 out of 10 at baseline to 1.2 out of 10 at 12 weeks (67% reduction; mean reduction 2.3, 95% CI 1.5-3.1, *P*<.001). At 6 months participants still expressed low interest in surgery (69% reduction; mean reduction: 2.4, 95% CI 1.6-3.2, *P*<.001). Of the 17 participants at high risk of surgery at baseline—defined as a surgery interest of 5 or higher—by week 12 only 3 remained at high risk. At 6 months, still only 3 remained at high risk for surgery, 2 of whom also were at high surgery risk at week 12, and 1 of whom had moved into the high-risk category between week 12 and 6 months.

#### Satisfaction

Participants expressed high satisfaction with the program. At week 12, on average participants rated the program 9.2 out of 10 (SD 1.3). By 6 months, the average rating was 9.3 (SD 1.1).

## Discussion

### Principal Findings

Although CKP is a common cause of severe chronic pain and disability affecting millions of individuals, accessible comprehensive treatment programs that address multiple components of care are lacking. The challenges to effectively delivering a program involving physical therapy, education, and psychosocial support are diverse and substantial—including time constraints on primary care appointments, paucity of reimbursement for education, and lack of awareness of the psychosocial risk factors that impact outcomes for CKP. Moreover, there are significant practical and cost barriers faced by the patient—such as traveling to physical therapy appointments, large patient costs, sourcing and paying for childcare, or having to seek out education and psychosocial support on their own. Finally, tracking outcomes and program adherence is difficult if not impossible in the traditional outpatient setting, and there is a distinct lack of technology-enabled solutions for patients. The results of this study demonstrated that the Hinge Health 12-week DCP for individuals with CKP produced clinically and statistically significant improvements in knee pain, stiffness, and function that lasted over a period of 6 months following initiation of the 12-week program, and were accompanied by a significant reduction in surgery interest as well as high satisfaction. Furthermore, the digitization of exercise therapy allowed for precise tracking of participation and adherence, showing that on average participants completed exercise therapy between 3 and 4 days each week.

Participants’ KOOS pain and function scores improved by clinically significant 16 and 10 points, respectively, at the end of the 12-week program. Similarly, VAS pain and stiffness scores improved by clinically significant 58% and 50% at the end of the 12-week program. These improvements are greater than or of similar magnitude to other treatment programs that have shown efficacy for CKP, including a 12-week graded physical activity exercise program which found improvements in WOMAC pain and function of 25% and 22%, respectively, immediately after program completion [39]; an 8-week exercise and education program which found improvements in WOMAC pain, stiffness, and function scores of 23%, 17%, and 23%, respectively, immediately after the completion of the program [40]; and a 6-week exercise, education, and self-management program which found improvements in WOMAC pain and function of 31% and 26%, respectively, immediately at the end of the program [10]. Deyle et al [41] found greater improvement in WOMAC score at the end of a 4-week program of manual therapy and supervised exercise (52%) versus a home-based exercise program (26%). However, the clinical intervention was more expensive than the home-based intervention and did not lead to better long-term outcomes [41], and the home-based intervention did not include any program components such as education or behavioral therapy which may improve long-term outcomes. The format of the program also did not allow the researchers to track adherence to the home exercise.

The clinically significant improvements in KOOS pain and function in this study were maintained at 6 months after starting the program, with improvements of 18 and 14 points, respectively. Similarly, the improvements in VAS pain and stiffness scores were maintained, with improvements of 60% and 58% at the 6-month timepoint, respectively. These results suggest strong maintenance of effect of the program. Similar long-term effects have been reported in other intervention programs of similar length [10,12,39-41], with clinical improvements reported to be maintained as long as 30 months after completing the programs. Although the long-term effect of the Hinge Health DCP, in particular the effect related to exercise, may in part be dependent on continued adherence to the program [42], the behavioral, educational, and psychosocial components of the program may improve the potential for long-term effects [10]. Furthermore, the comprehensive conservative care program incorporating exercise may also influence the need for future surgical treatments, as a previous treatment program incorporating exercise and manual physical therapy found a 75% reduction in TKA after participation in the program [12]. Similarly, comprehensive pain management programs for chronic back pain demonstrate a reduced need for surgery of 67% as compared with alternative medical care [6]. Surgical interventions such as TKA are effective at improving pain and symptoms following surgery, with studies finding between approximately 50% and 75% of patients experience improvement after surgery [43,44]. However, even in individuals with CKP that have all indications to warrant surgery, afflicted individuals are often reluctant to consider invasive surgical procedures, with data showing only 15-32% are willing to consider surgery for their knee pain [45,46]. In this study, surgery interest significantly decreased over the course of the 12-week program, with no participant increasing in intent for surgery. These improvements in pain and function could be maintained over the long-term, thereby circumventing surgery and its cost. However, the follow-up period of this study was too short to draw a definitive conclusion on the matter, and future research will be needed to more fully understand the economic effects of the program.

### Strengths and Limitations

The results of this study demonstrate that the Hinge Health DCP shows promise for providing participants with a program to effectively manage their CKP condition. However, this study has several limitations. This was a single-arm study without blinding of the participants, and thus any placebo effect, for example, due to simply being accepted into the program, or regression to the mean was not able to be evaluated. Future work with a more rigorous study design such as a randomized, controlled trial as compared with standard care or multiple baseline trial will be needed to better understand the effect of the program as compared with standard care. Although the sample size was relatively small, the results demonstrated large effect sizes for primary outcomes which showed highly significant results and should be confirmed in larger future studies.

The study enrolled participants with self-reported CKP, but did not require a physician-diagnosis of knee OA. However, our recruitment questionnaire utilized questions specific to clinical diagnosis for knee OA derived from the American College of Rheumatology criteria for OA of the knee [2], and our inclusion criteria are similar to those of other knee OA studies [12,39-41]. Furthermore, participants included in this study showed typical demographics and characteristics of people living with CKP (Table 1). Our participants were predominantly female, and although a higher prevalence of knee OA and knee pain are reported in female versus male [47,48], future work should include a larger male participant population to better understand potential differences in program response due to sex.

Study results showed good subject engagement with exercise and education. However, due to the comprehensive nature of the program, it is not possible to determine if all components of the program are integral to the study results. As shown in Figure 4, we noted a substantial drop in knee pain and stiffness between baseline (screening) and the first VAS score reported, potentially as a positive consequence of the exercises performed as part of onboarding, regression to the mean, and perceived improvements due to the positive news of being accepted onto the program. To confirm that the program achieved improved outcomes not just between baseline and the first VAS, we also compared the average VAS ratings in weeks 1-4 of the program against those in weeks 9-12, and observed highly significant reductions in pain (9.3 points, 95% CI 5.7-12.8, *P*<.001) and stiffness (8.4 points, 95% CI 4.8-12.0, *P*<.001). Deyle et al [12] also noted a rapid reduction in symptoms of 20-40% after only a few treatment sessions, which was attributed to improvement from the initial therapy. Although other treatments of similar duration have found lasting effects [10,12,39,40,49], the relatively short time frame of this study, to 3 months follow-up after completion of the program, or 6 months after enrollment, requires future work to evaluate the potential of the program for long-term improvement in symptoms.

### Conclusions

The results of this study demonstrated clinically and statistically significant improvements in pain, function, and stiffness following a 12-week digitally based program designed to address multiple components of care for CKP. Although the initial results with this program are promising, future research will be needed to understand the long-term effects of the program. Due to the adaptability of the system, future work may also investigate the effect of a similar program on other chronic pain conditions such as lower back pain.

In conclusion, the results of this pilot study of the 12-week digital Hinge Health DCP demonstrate improvements in knee pain, stiffness, and function which were maintained to 6 months after enrollment into the program. The program greatly reduced surgery interest in participants, providing strong evidence that the program may be an effective intervention to delay or significantly reduce the incidence of more invasive and costly treatments for CKP such as surgery.

## Abbreviations

CBT: cognitive behavioral therapy
CKP: chronic knee pain
DCP: digital care program
HADS: Hospital Anxiety and Depression Scale
KOOS: Knee Injury and Osteoarthritis Outcome Score
KOOS-PS: Knee Injury and Osteoarthritis Outcome Score-Physical Function Short Form
OA: osteoarthritis
PROM: patient-reported outcome measure
TKA: total knee arthroplasty
VAS: visual analog scale
